# Assessing the contribution of diazotrophs to microbial Fe uptake using a group specific approach in the Western Tropical South Pacific Ocean

**DOI:** 10.1038/s43705-022-00122-7

**Published:** 2022-04-27

**Authors:** C. Lory, F. Van Wambeke, M. Fourquez, A. Barani, C. Guieu, C. Tilliette, D. Marie, S. Nunige, I. Berman-Frank, S. Bonnet

**Affiliations:** 1 Aix Marseille Université, Université de Toulon, CNRS, IRD, MIO, Marseille, France; 2 Sorbonne Université, CNRS, Laboratoire d’Océanographie de Villefranche, LOV, F-06230 Villefranche-sur-Mer, France; 3 Sorbonne Université, CNRS, Station Biologique de Roscoff, Roscoff, France; 4 Department of Marine Biology, The Leon H. Charney School of Marine Sciences, University of Haifa, Haifa, Israel

**Keywords:** Biogeochemistry, Biogeochemistry

## Abstract

Diazotrophs are often limited by iron (Fe) availability in the oligotrophic ocean. The Western Tropical South Pacific (WTSP) ocean has been suggested as an intense N_2_ fixation area due to Fe fertilizations through shallow hydrothermal activity. Yet, the Fe demand of diazotrophs in their natural habitat, where they cohabit with other microbial organisms also requiring Fe, remains unknown. Here we develop and apply a method consisting of coupling ^55^Fe uptake experiments with cell-sorting by flow cytometry, and provide group-specific rates of *in situ* Fe uptake by the microbial community in the WTSP, in addition to bulk and size fractionation rates. We reveal that the diazotrophs *Crocosphaera watsonii* and *Trichodesmium* contribute substantially to the bulk *in situ* Fe uptake (~33% on average over the studied area), despite being numerically less abundant compared to the rest of the planktonic community. *Trichodesmium* had the highest cell-specific Fe uptake rates, followed by *C. watsonii*, picoeukaryotes, *Prochlorococcus*, *Synechococcus* and finally heterotrophic bacteria. Calculated Fe:C quotas were higher (by 2 to 52-fold) for both studied diazotrophs compared to those of the non-diazotrophic plankton, reflecting their high intrinsic Fe demand. This translates into a diazotroph biogeographical distribution that appears to be influenced by ambient dissolved Fe concentrations in the WTSP. Despite having low cell-specific uptake rates, *Prochlorococcus* and heterotrophic bacteria were largely the main contributors to the bulk Fe uptake (~23% and ~12%, respectively). Overall, this group-specific approach increases our ability to examine the ecophysiological role of functional groups, including those of less abundant and/or less active microbes.

## Introduction

Planktonic dinitrogen (N_2_) fixation is the major source of new N to the surface ocean (~106–120 Tg N y^−1^; [1, 2]), and sustains most new primary production (PP) and organic matter export in low nutrient low chlorophyll (LNLC) ecosystems [3–6]. N_2_ fixation is performed by diazotrophs, prokaryotic organisms composed of filamentous or unicellular photoautotrophic cyanobacteria (UCYN; [7]), and several classes of non-cyanobacterial diazotrophs [8]. The growth of photoautotrophic diazotrophs is regulated by several macro- and micro-nutrients, with phosphorus (P) and iron (Fe) identified as essential regulators of N_2_ fixation, photosynthesis, and growth in different oceanic areas [9–15]. N_2_ fixation especially is catalyzed by a metalloenzyme complex (the nitrogenase reductase) containing three metal prosthetic groups highly enriched in Fe, that impose a high Fe demand for diazotrophs [16–18].

Monospecific culture experiments indicate that photosynthetic activity, N_2_ fixation and thus Fe requirements vary among the major diazotroph groups [19]. The filamentous cyanobacterium *Trichodesmium* spp. (hereafter named *Trichodesmium*) performs oxygenic photosynthesis and N_2_ fixation with spatial and temporal segregation during daylight [20]. Both processes require Fe-rich proteins [21] and under Fe limitation, *Trichodesmium* allocates most of its intracellular Fe to photosynthesis and down-regulates N_2_ fixation [21, 22]. When Fe limitation is alleviated (and other essential nutrients replete), *Trichodesmium* has an efficient Fe uptake system and can store excess Fe [23]. In contrast, UCYN from groups B and C fix N_2_ at night and perform photosynthesis during the day. For *Crocosphaera watsonii* (UCYN-B), this diel cycle imposes a daily synthesis and degradation of the Fe-rich metalloproteins involved in these two processes [24], leading to a 40% reduction of cellular Fe requirements compared to organisms that do not [22, 24]. Cultured *C. watsonii* may also decrease its cell-size to optimize N_2_ fixation capacities and photosynthetic activity under Fe deprivation [25]. These combined observations suggest that UCYN would be better adapted to Fe-depleted waters than *Trichodesmium*. If this assumption is correct, ambient dissolved Fe (dFe) conditions may play a central role in defining the biogeographical distribution of photo-autotrophic diazotrophs in the ocean. However, in situ Fe uptake kinetics of natural diazotroph populations remain sparse [26–28], and no in situ data are available to date for UCYN. These knowledge gaps prohibit an assessment of how Fe availability structures the present distribution of diazotrophs in the ocean.

The acquisition of Fe is influenced by several factors such as the Fe uptake pathways and cellular requirements that differ among marine microbes [29, 30]. While Fe bioavailability is difficult to constraint, measuring phytoplankton Fe uptake kinetics can provide insights on the contrasted Fe requirements between species [31, 32]. In particular, Fe uptake rates from cultured cyanobacteria and eukaryotes have demonstrated that small organisms are more efficient than larger one in meeting their Fe demand due to their high surface:volume ratios [33]. The nature of the dFe pool also influence the Fe uptake rates as eukaryotic phytoplankton internalize Fe-complexed to siderophores at faster rates than cyanobacteria [34]. In the natural environment, diazotrophs are mixed with other planktonic groups that also require Fe, potentially competing for this micronutrient. Yet, quantitative information on the in situ Fe uptake by specific groups of the microbial assemblage remains sparse [35, 36] and new approaches are needed to evaluate how different microbes, including diazotrophs, access and compete for this essential micronutrient.

Additionally, contrary to HNLC waters, the biological Fe demand in LNLC water is poorly surveyed despite reports that Fe limits N_2_ fixation in several areas including the oligotrophic North [37] and South Pacific oceans [38], and the eastern tropical North Atlantic [9]. In particular, the Western Tropical South Pacific (WTSP) ocean that stretches from Australia to the Western boundary of the South Pacific Gyre has received little attention, although *Trichodesmium* blooms were historically observed by satellite imagery [39]. More recent cruises confirmed high diazotroph abundances [40–42] in this region qualified as a global hot-spot of N_2_ fixation (>600 µmol N m^−2^ d^−1^, [43]). The ecological success of diazotrophs in this region has been attributed to a combination of favorable factors such as Fe fertilization processes through shallow (<500 m) underwater volcanoes associated with hydrothermal activity [44], high sea surface temperature (>25 °C), and sufficient phosphate availability [43, 45, 46]. Yet, the quantification of in situ Fe-uptake rates by the whole planktonic community in general, and by diazotrophs in particular, is still lacking in this region.

Here we investigated the potential link between the biological Fe uptake and the diazotroph activity and distribution in the WTSP. We conducted isotopic ^55^Fe and ^15^N_2_ incubation experiments across dFe gradients, followed by size fractionation. Furthermore, we developed and applied a method consisting of coupling ^55^Fe uptake experiments with cell-sorting by flow cytometry and provide a precise assessment of in situ Fe uptake by the different members of the microbial community, including photoautotrophic diazotrophs. We compared group-specific Fe uptake rates of the in situ photoautotrophic diazotroph community with those of the dominant surrounding microorganisms. Finally, we used a dFe bioavailability proxy to assess the bioavailability of the WTSP seawater for different cyanobacteria (*Trichodesmium*, *C. watsonii*, *Synechococcus*, *Prochlorococcus*), picoeukaryotes, and heterotrophic bacteria (HB).

## Materials and methods

### Sampling procedures

**Fig. 1:**
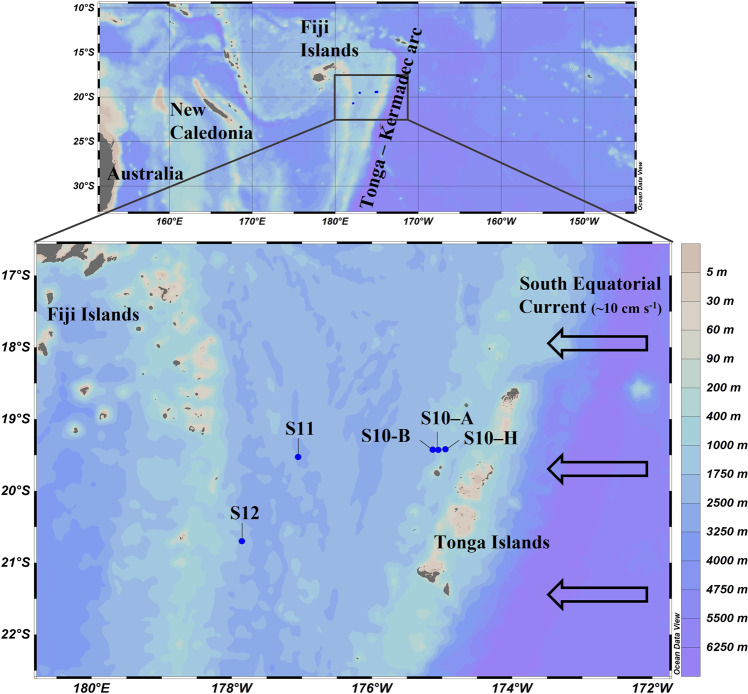
Map of the study area in the WTSP showing the stations sampled for Fe uptake experiments. Near the Tonga volcanic arc, we sampled 3 stations (Tonga arc stations) along a spatial gradient going from the shallowest station (S10-H, where hydrothermal signals were recorded) to two farther stations located 5 NM (S10-A) and 10 NM westward (S10-B ~2000 m). S11 and S12 were situated further west (~90 NM from the Tonga volcanic arc, named western stations) in the Lau basin. Arrows indicate the westward flowing South Equatorial Current (7–10 cm s^−1^ [50]). The base map shows the bathymetry in meters.

Samples were collected during the GEOTRACES-endorsed TONGA cruise (doi:10.17600/18000884) onboard the R/V L’Atalante in October–December 2019 (beginning of austral summer). Surface seawater (5 m depth) was collected at five stations (Fig. 1) under trace metal clean conditions from Teflon-coated 12 L GoFlo bottles mounted on a titanium Trace Metal clean Rosette (TMR, General Oceanics Inc. Model 1018 Intelligent Rosette). In line with previous cruises in this region [44, 47], chemical and physical anomalies were measured at shallow depths (~300 m) in the vicinity of the Tonga volcanic arc, revealing hydrothermal activity [48]. Our sampling strategy consisted of sampling along a spatial gradient going from the shallowest station S10-H (~300 m), where hydrothermal signals (acoustic anomalies) were recorded, to two farther stations located 5 nautical miles (NM) (S10-A, ~700 m), and 10 NM (S10-B, ~2000 m) from S10-H westward, i.e. in the main current direction of the South Equatorial Current (Fig. 1, hereafter named ‘Tonga arc stations’) [48]. Two additional stations (S11 and S12) were sampled further west (~90 NM from the Tonga volcanic arc) in the Lau basin (hereafter named ‘western stations’).

All samples were collected inside a clean container in polycarbonate (PC) bottles, previously acid-washed according to GEOTRACES protocols (GEOTRACES cookbook) [49]. The essential methodology is included in the following section, while more details are reported in the Supplementary materials (S).

### Fe uptake rates measurements

The first set of experiments (‘Size fractionation’ SF-experiments) consisted of measuring the Fe uptake rates of the pico-, nano- and microplanktonic microbial size fractions at all stations (Figs. 1 and S1). All manipulations were conducted under a laminar flow hood. Triplicate PC-bottles were filled with 500 mL surface seawater and spiked with a working solution of ^55^FeCl_3_ diluted in 2.5 × 10^−3^ M Ultrapure HCl (Perkin Elmer, specific activity 6.21 × 10^3^ Ci mol^−1^) to reach a final concentration of 0.2 nM ^55^Fe which accounted for 11–45% of total dFe. Bottles were incubated for 24 h under in situ-simulated conditions in on-deck incubators covered by blue screening and connected to surface circulating seawater. Samples were then sequentially filtered through a stack of 10 µm, 2 µm, and 0.2 µm pore size PC filters (47 mm diameter, Nuclepore). To account for the sole “biological” intracellular fraction of ^55^Fe [51], each filter was washed twice with 6 mL of a Ti(III)-citrate-EDTA reagent [52, 53] for 2 min to remove extracellular adsorbed Fe and Fe (oxy)hydroxide precipitates, and subsequently rinsed three times for 1 min with 5 mL of 0.2 µm filtered trace metal clean sampled-seawater. Filters were then placed in scintillation plastic vials with 5 mL of scintillation cocktail (UltimaGold MV, Perkin Elmer), that were agitated before the radioactivity was counted onboard with a Hidex® 300SL scintillation counter. For each station, one procedural blank was measured for each size-fraction to account for background ^55^Fe uptake (see blank measurements and calculations in the Supplementary materials). After subtracting the blank from the counts per minute to the filter (see Eq. S1.a), the total radioactivity on 10 µm, 2 µm, and 0.2 µm filters represent the contribution of micro-, nano-, and picoplankton to intracellular ^55^Fe uptake, respectively.

For the second set of experiments (‘Group Specific’ GS-experiments), triplicate 2 L PC-bottles were spiked with 0.2 nM final concentration of ^55^Fe and incubated the same way as the SF-experiment (Fig. S1). Samples were then sequentially filtered through 10 µm PC filters to recover large-size phytoplankton (mainly *Trichodesmium*) and 0.2 µm filters to recover small size cyanobacterial diazotrophs and other planktonic organisms for further cell-sorting. Each filter was placed in a cryotube filled with 4.5 mL of 0.2 µm-filtered seawater and paraformaldehyde (2% final concentration) for 15 min in the dark. Cryotubes were then vortexed to detach the cells from the filter [54] and stored at −80 °C until processed onshore.

Cell-sorting from the concentrate of the <10 µm fraction (0.2 µm filter) was performed for *C. watsonii*-like (UCYN-B), *Synechococcus* spp. cell-like (hereafter called *Synechococcus*), *Prochlorococcus* spp. cell-like (hereafter called *Prochlorococcus*), picoeukaryotes, and HB by sorting cells with low-nucleic acid content (LNA) and high-nucleic acid content (HNA) on a Becton Dickinson Influx Mariner (BD Biosciences, Franklin Lakes, NJ) high-speed cell sorter, at the Regional Flow Cytometry Platform for Microbiology as described in Bonnet et al[54]. and Berthelot et al[55]. (Cytograms in Fig. S2). All sorted organisms were then deposited on 0.2 µm filters and rinsed with the same protocol as described in the SF-experiment to remove extracellular Fe.

A minimum threshold in the number of sorted cells was required to detect a radioactive signal at least two-fold higher than that of the blank filter: 200 × 10^3^ cells of *C. watsonii*, 1.5 × 10^6^ cells of *Synechococcus*, 0.1 × 10^6^ cells of *Prochlorococcus*, 30 × 10^3^ cells of picoeukaryotes and 5 × 10^6^ cells of HB and we subtracted blanks specific to each organism (see blanks details in the Supplementary materials). From all studied stations, we were able to sort enough cells for each targeted organism at S10-B, S10-A, and S10-H, except for *Prochlorococcus* at S10-B. For *Trichodesmium*, cryotubes containing the concentrate of the >10 µm fraction were filtered on 20 µm filters and filaments were enumerated for each station by counting the whole filter using epifluorescence microscopy (200 filaments minimum were needed for significant ^55^Fe detection). To ensure that measured Fe uptake rates were attributed to *Trichodesmium*, we checked by microscopy that other groups (such as diatoms) were not present on these filters.

### Dissolved Fe concentrations

Samples for dFe concentrations were directly filtered from the GoFlo bottles using a 0.45-µm polyethersulfone filter (SuporR) and acidified within 24 h of collection with ultrapure HCl (Merck, 0.2% final concentration, pH 1.7). DFe concentrations were determined using Flow Injection Analysis with online preconcentration and chemiluminescence detection as described in Blain et al. [56]. The D1 SAFe seawater standard was analyzed to monitor the consistency of the method, and an internal acidified seawater standard was measured every day to verify the stability of the analysis.

### Calculations

For the SF-experiment, Fe uptake rates (in mol Fe L^−1^ d^−1^) for each size fraction (*ρ*_pico_, *ρ*_nano_, and *ρ*_micro_) were calculated based on the activities recorded on each filter as a function of incubation time and volume filtered, using the equations from Sarthou et al. [57] (see Eq. S1a, b). Fe uptake rates of the bulk (*ρ*_Bulk_) was the sum of *ρ*_pico_, *ρ*_nano_, and *ρ*_micro_ (Eq. S1c). Group-specific Fe uptake rates (*ρ*_GS_ in mol Fe cell^−1^ d^−1^) were calculated using the same formula as for *ρ*_SF_ but by accounting for the number of cells sorted (see Eq. S2a–c). Corrections for blanks (see Supplementary materials), radioactive decay and dFe concentration of seawater (in nM) at each station were applied for both experiments. The contribution (in %) of each sorted organism was calculated as described in Eq. S3a, b. For the cell-surface area (S.A.) calculations, we used the geometric models from Sun et al. [58] and considered a spherical shape for unicellular organisms and a cylinder girdle for *Trichodesmium* (details in the legend of Table 2).

To compare group-specific Fe uptake with different dFe concentrations, we calculated the apparent Fe uptake rate constant *k*_in-app_ (L cell^−1^ d^−1^; Eq. S4, [34]) for each sorted organism, using the term “app” to reflect the probable assemblage of natural Fe complexes during the uptake experiment [59]. To calculate the *k*_in-app_, we assumed that phytoplankton were accessing Fe mostly from the organic pool [32, 60] and that Fe uptake was linear over the incubation experiment. For the latter assumption, we could not measure group-specific Fe uptake kinetics to ensure a constant uptake rate throughout the incubation. However, for the uptake to remain linear, the phytoplankton should not be affected by the depletion of the dFe throughout the incubation experiment [59], thereby the Fe acquired by the cells should be substantially lower than the pool of dFe available. This criterion was verified as we estimated that all acquired Fe by the cells accounted, at most, for 2% of the total dFe concentration. Note that we did not include S10-A data as dFe concentration was too high (1.56 nM) to meet the criterion defined by Shaked et al. [61], which considers that dFe concentration must be <0.6 nM and log_10_(N µM/Fe nM) > 1 for the phytoplankton to be Fe-limited.

### Environmental parameters, dissolved and particulate stocks

Sea surface temperature was measured using a Seabird 911 CTD (conductivity, temperature, depth) at all stations. Samples for the quantification of nitrate (NO_3_^−^) and dissolved inorganic phosphorus (DIP) concentrations were filtered (Sartorius Sartobran-P-capsule 0.2 μm filter) and analyzed by standard colorimetric techniques on a AA3 AutoAnalyzer (Seal-Analytical) [62] (detection limits were 0.05 µM for NO_3_^−^ and 0.02 µM for DIP). Samples for dissolved organic carbon (DOC) were filtered through precombusted (24 h, 450 °C) glass fiber filters (Whatman GF/F, 25 mm), acidified with sulfuric acid and analyzed by high-temperature catalytic oxidation on a Shimadzu TOC-L analyzer (protocol was adapted from Sohrin et al. [63]). Particulate organic phosphorus (POP) content of the three size fractions was assessed for further normalization of Fe uptake rates. Surface seawater from the same depth and rosette as for the Fe uptake experiments was sequentially filtered through 10 µm, 2 µm, and 0.2 µm PC filters, stored at −20 °C and analyzed onshore according to the wet oxidation protocol described in [64]. Bulk particulate organic carbon (POC) concentrations were measured by filtering 4.5 L of seawater onto precombusted GF/F filters (450 °C, 4 h) that were stored at −20 °C until analysis by continuous flow isotope ratio mass spectrometry coupled to an elemental analyzer (EA-IRMS, Integra-2, SerCon Ltd). To accurately determine the cellular carbon content of *C. watsonii*, we sorted and filtered 500,000 additional cells at S10-B and analyzed them by EA-IRMS. For other organisms, we used cell-to-carbon conversion factors based on the literature (details in legend of Table 2).

### Determination of primary production and N2 fixation rates

Seawater for N_2_ fixation and PP rates measurements was sampled under trace metal clean conditions inside the clean container at the same stations and depth as Fe uptake rates by using the dual isotopic labeling ^13^C and ^15^N_2_ tracer method [65]. The ^15^N_2_ bubble technique was intentionally chosen to avoid any potential overestimation due to trace metal and dissolved organic matter (DOM) contaminations that might be associated with the preparation of the ^15^N_2_-enriched seawater [66, 67] or with the number of manipulations associated with the bubble release method [66], as Fe and DOM have been found to control N_2_ fixation or *nifH* gene expression in this region [68, 69]. However, the ^15^N/^14^N ratio of the N_2_ pool available for N_2_ fixation (the term AN_2_ used in Montoya et al. [70]) was measured in all incubation bottles by membrane inlet mass spectrometry (MIMS) to ensure accurate rate calculations, but we cannot exclude any potential underestimation of the N_2_ fixation rates reported here [71].

After labeling with H^13^CO_3_^−^ (98,9  atom%, Cambridge isotopes, 9‰ final enrichment) and 1 mL of ^15^N_2_ per liter of seawater (98.9 atom% ^15^N, Eurisotop), bottles were incubated under in situ-simulated conditions for 24 h and filtered as described in [6]. The ^13^C/^12^C and ^15^N/^14^N ratios were determined using EA-IRMS (Sercon Integra-2), with accuracy control of the system using International Atomic Energy Agency reference materials (AIEA-N-1 and IAEA-310A).

### Determination of pico-, nanoplankton, and diazotroph abundances

At the same stations as for the Fe uptake experiment, seawater was sampled to determine in situ abundances of *Synechococcus*, *Prochlorococcus*, picoeukaryotes and HB (sum of HNA and LNA) using a Becton Dickinson Facs Canto II as described in Marie et al. [72]. The abundances of *C. watsonii* and *Trichodesmium* were quantified using quantitative PCR (qPCR) analysis of the *nifH* gene. 2.3 L of surface seawater (5 m) were filtered onto 0.2 µm Supor filters at each station and DNA extracted and TaqMAN qPCR assays performed as previously described in Stenegren et al. [73]. Note that UCYN-A and non-cyanobacterial diazotrophs were also quantified by qPCR but were not studied here.

As the quantification of *Trichodesmium* by qPCR has been seen to be overestimated by qPCR assays [74] due to its polyploidy [75], *Trichodesmium* were also enumerated by microscopy. In all, 2.3 L of surface seawater collected at each station from the same TMR rosette used for the incubation experiment were filtered on 10 µm and 2 µm PC filters, fixed with paraformaldehyde (2% final concentration) for at least 15 min and stored at −20 °C. Abundances were determined by counting the filaments on the filter (minimum 200 filaments) using an epifluorescence microscope (Zeiss Axioplan, Jana, Germany) fitted with a green (510–560 nm) excitation filter.

## Results

### Environmental conditions in the study area

Surface seawater temperature was relatively constant among sampled stations (25–27 °C) and surface (5 m) dFe concentrations ranged from 0.33 nM at western stations (S11 and S12) to 1.56 nM at the Tonga arc station S10-A (Table 1). NO_3_^−^ were below quantification limits at all stations and DIP concentrations ranged from below quantification limit (at western stations S11 and S12) to 0.06 µM (at S10 stations) (Table S1). DOC concentrations varied from 70 to 80 µM. At all stations, the microbial community was dominated by HB (6–10 × 10^5^ cells mL^−1^), followed by *Prochlorococcus* (0.7–5 × 10^4^ cells mL^−1^), *Synechococcus* (2.5–10 × 10^3^ cells mL^−1^), and picoeukaryotes (250–500 cells mL^−1^) (Table S1). The photoautotrophic cyanobacterial diazotroph community was dominated by *Trichodesmium* (4–32 × 10^6^ *nifH* copies L^−1^) and *C. watsonii* (4.8 × 10^4^ to 4.0 × 10^6^ *nifH* gene copies L^−1^) (Table S1). In terms of C biomass, the community was dominated by *Trichodesmium* (5–7 µmol C L^−1^), followed by *C. watsonii* (0.05–3 µmol C L^−1^) and by HB (0.6–1 µmol C L^−1^), while *Synechococcus, Prochlorococcus* and picoeukaryotes contributed for <0.2 µmol C L^−1^ (Table S1).

**Table 1:** dFe concentrations, bulk, POC-normalized and size-fraction Fe uptake rates measured at each station.

	S12	S11	S10 - B	S10–A	S10 - H
dFe	0.24 ± 0.01	0.41 ± 0.01	0.49 ± 0.01	1.56 ± 0.01	0.35 ± 0.02
(nM)					
*ρ*_Bulk_	6.5 ± 4.6	6.5 ± 3.9	17 ± 14	19 ± 11.5	15 ± 7.4
(pmol Fe L^−1^ d^−1^)					
*ρ*_Bulk_: POC	1.2 ± 0.9	1.4 ± 0.9	3.5 ± 2.9	n/a	3.3 ± 1.7
(µmol Fe mol C^−1^ d^−1^)					
*ρ*_micro_	2.6 ± 1.9	2.3 ± 1	3.9 ± 2.5	3.6 ± 1.9	3.7 ± 1.9
(pmol Fe L^−1^ d^−1^)					
*ρ*_nano_	1.1 ± 0.7	0.9 ± 0.6	2.4±1.8	5.1 ± 2.6	3.3 ± 2
(pmol Fe L^−1^ d^−1^)					
*ρ*_pico_	2.8 ± 2	3.2 ± 2.3	11 ± 9.4	10 ± 7	7.9 ± 3.6
(pmol Fe L^−1^ d^−1^)					

N_2_ fixation rates varied between 15 ± 9 and 65 ± 15.5 nmol N L^−1^ d^−1^ and PP varied between 0.6 ± 0.004 and 2.5 ± 0.7 µmol C L^−1^ d^−1^ with highest rates measured at S10-B for both N_2_ fixation and PP (Table S1).

### Bulk and size-fractionated Fe uptake rates

Bulk Fe uptake rates (*ρ*_Bulk_) ranged from 6.5 to 19.1 pmol Fe L^−1^ d^−1^. They were significantly higher (by 2.7-fold) at the Tonga arc stations (S10 stations; 15–19 pmol Fe L^−1^ d^−1^) as compared to the western stations (S11 and S12; 6.5 pmol Fe L^−1^ d^−1^; *p* < 0.05 non-parametric Mann–Whitney test; Table 1 and detailed data in Table S2). A similar trend was observed for POC-normalized Fe uptake rates, with a 2.6-fold factor between both areas (averaged 3.4 µmol Fe mol C^−1^  d^−1^ and 1.3 µmol Fe mol C^−1^ d^−1^ at the Tonga arc and western stations, respectively, *p* < 0.05 non-parametric Mann–Whitney test). Bulk Fe uptake rates correlated with N_2_ fixation rates over the studied transect (*n* = 4, *r* = 0.962, *p* < 0.05 Pearson correlation) but not with PP (*n* = 4, *r* = 0.509, *p* > 0.05 Pearson correlation) (N_2_ fixation rates and PP in Table S1).

**Fig. 2:**
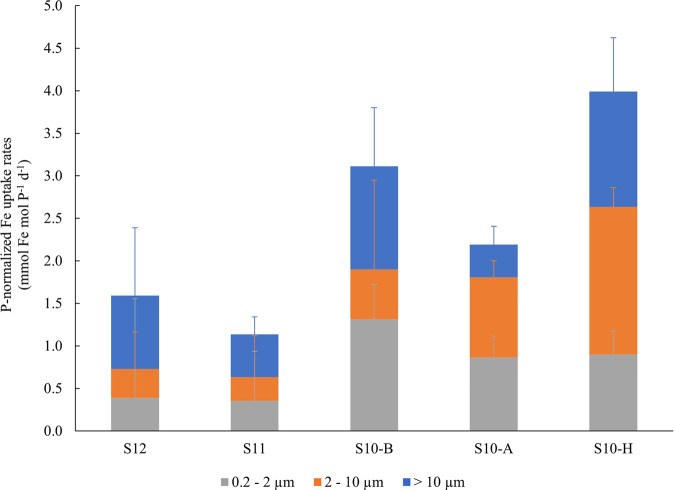
Fe demand of the microbial community. P-normalized Fe uptake rates for the micro- (>10 µm), nano- (2–10 µm), and picoplankton (0.2–2 µm) fractions measured at each station at 5 m depth. Error bars indicate standard deviations (*n* = 3).

At all stations, the picoplanktonic fraction (0.2–2 µm) accounted for the highest Fe uptake (*ρ*_pico_ in pmol Fe L^−1^ d^−1^) (~52 ± 7%) compared to the nano- (2–10 µm; ~19 ± 5%) and microplanktonic (>10 µm; ~28 ± 9%) size-fractions (*p* < 0.05, non-parametric Mann–Whitney test) (Table 1). To account for the differences in the biomass among microbial size-fractions, the Fe uptake rates of each size-fraction were normalized to their respective POP content at the same station and depth (Fig. 2). When normalized by POP, the contribution of the picoplankton size-fraction (0.2–2 µm) was mitigated to meet ~32 ± 9% on average over all stations, similar to that of the nanoplankton size class (2–10 µm) (~30 ± 12%) and microplanktonic size class (>10 µm) (~38 ± 14%) fractions (*p* > 0.05, non-parametric Mann–Whitney test).

### Cell-specific Fe uptake rates

**Fig. 3:**
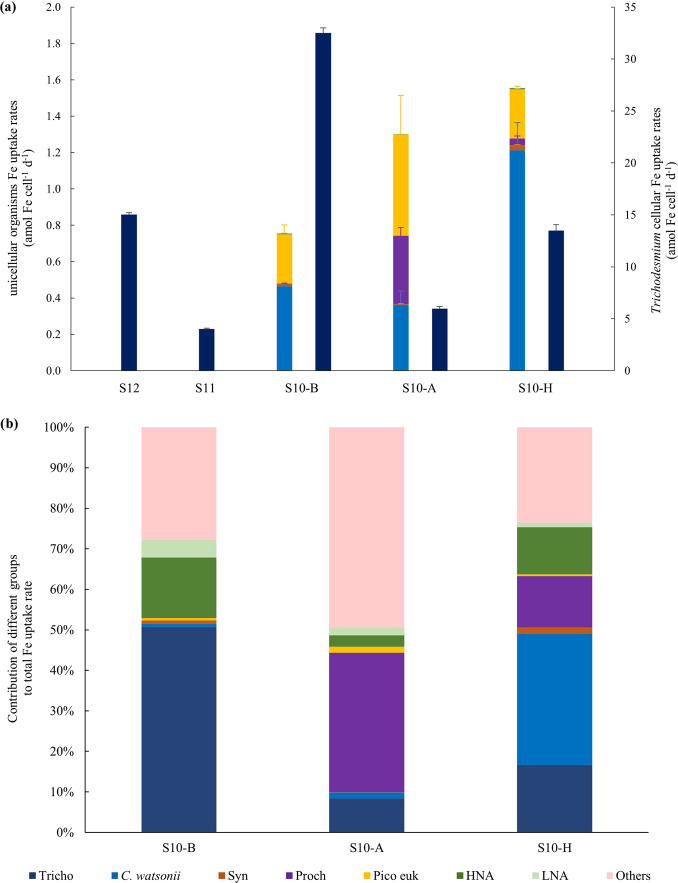
Group-specific Fe demand. **a** Cellular Fe uptake rates for *Trichodesmium* (Tricho) (right scale, second bar) and unicellular organisms (left scale, first bar): *C. watsonii*, *Synechococcus* (Syn), *Prochlorococcus* (Proch), picoeukaryotes (Pico euk), HNA and LNA. Note that at S11 and S12, not enough cells were recovered for sorting unicellular organisms by flow cytometry and not enough *Prochlorococcus* at S10-B (see text). Error bars are calculated from triplicate counting of the same sample as not enough cells were recovered for triplicate samples measurements. Each group is labeled in a unique color that is consistent across all panels. **b** Relative contribution (%) of each sorted group from the GS-experiment to the bulk Fe uptake rates, from the SF-experiment.

To facilitate the comparison between unicellular and filamentous species, we report Fe uptake rates per cell for *Trichodesmium* assuming that a filament is composed of 100 cells, based on our direct counts (110 ± 45 cells trichome^−1^), in line with the literature [76, 79]. *Trichodesmium* had the highest uptake rates per cell (4.02–32.51 amol Fe cell^−1^ d^−1^), followed by *C. watsonii* (0.46–1.21 amol Fe cell^−1^ d^−1^), picoeukaryotes (0.27–0.56 amol Fe cell^−1^ d^−1^), *Prochlorococcus* (0.05–0.38 amol Fe cell^−1^ d^−1^) and *Synechococcus* (0.005–0.027 amol Fe cell^−1^ d^−1^) (Fig. 3a and detailed data in Table S3). HB exhibited one to two orders of magnitude lower cellular Fe uptake rates compared to other groups (0.001–0.003 amol Fe cell^−1^ d^−1^). For all sorted organisms, the highest Fe uptake rates per cell were measured at S10-H and S10-B, excluding *Prochlorococcus* (note that not enough *Prochlorococcus* could be sorted at S10-B). It has to be noted that *Trichodesmium* were filtered on 10 µm filters, and epibiotic bacteria might have been retained on the filters. By considering ~500 bacteria per filament of *Trichodesmium* [28], the contribution of Fe uptake by bacteria (*ρ*_HB_, in mol Fe cell^−1^ d^−1^) was 1000-fold lower than that of one filament and is thus considered negligible.

On the cell surface, the number of Fe transporters is constrained by the size of the transporters and the available membrane space allocated for Fe acquisition [30, 80]. Normalizing Fe uptake rates per cell by their surface area (S.A.) therefore better reflects the differences between organisms to uptake Fe [34, 59]. The highest S.A. normalized Fe uptake rates (Table 2) were measured for *Trichodesmium*, *Prochlorococcus* and picoeukaryotes, while *C. watsonii*, *Synechococcus* and HB exhibited rates lower by one to two orders of magnitude.

**Table 2:** Cell surface area (S.A.), C content, S.A.-normalized Fe uptake rates, C-normalized Fe uptake rates, and cellular Fe uptake rate constant (kin-app) for each sorted microorganism.

	S.A.	C content	S.A.-normalized Fe-uptake rates			C-normalized Fe-uptake rates			*k*_in-app_	
	(µm^2^ cell^−1^)	(mol C cell^−1^)	(×10^−3^ amol Fe µm^−2^ d^−1^)			(µmol Fe mol C^−1^ d^−1^)^a^			(L cell^−1^ d^−1^)	
			S10-B	S10-A	S10-H	S10-B	S10-A	S10-H	S10-B	S10-H
*Trichodesmium*	14.176^b^	(5.41 ± 2.66) × 10^−12a^	229 ± 3.4	42 ± 1.5	95 ± 4	1.3 ± 0.02	0.2 ± 0.01	0.5 ± 0.02	3.8 × 10^−6^	1.9 × 10^−6^
*C. watsonii*	78.54	(7.31 ± 0.003) × 10^−13^	5.9 ± 0.1	4.6 ± 4.0	15.4 ± 13	0.6 ± 0.05	0.5 ± 0.1	1.7 ± 0.2	5.5 × 10^−10^	1.7 × 10^−9^
*Synechococcus*	3.142	(2.12 ± 0.36) × 10^−14^	4.9 ± 2.6	1.6 ± 0.8	8.7 ± 1.8	0.7 ± 0.4	0.2 ± 0.1	1.3 ± 0.3	1. × 10^−11^	3.9 × 10^−11^
*Prochlorococcus*	0.785	(3 ± 0.52) × 10^−15^	n/a	244 ± 30	23.6 ± 9	n/a	125 ± 28	12 ± 5.1	n/a	5.1 × 10^−11^
Pico-eukaryotes	2.011	(2.16 ± 1.48) × 10^−13^	134 ± 26	277 ± 21	135 ± 9	1.2 ± 0.4	2.6 ± 1.2	1.3 ± 0.4	3.2 × 10^−10^	3. × 10^−10^
HNA	0.283	(1.04 ± 0.52) × 10^−15^	16 ± 4.0	5.1 ± 2.1	18.8 ± 3.2	4.4 ± 2.5	1.4 ± 0.9	5.1 ± 2.8	5.3 × 10^−12^	7.5 × 10^−12^
LNA	0.283	(1.04 ± 0.52) × 10^−15^	7.0 ± 5.6	2.7 ± 1.1	2.0 ± 0.7	1.9 ± 1.8	0.7 ± 0.5	0.5 ± 0.3	2.3 × 10^−12^	8.0 × 10^−13^

S.A. were calculated using an averaged length of 900 µm and width of 5 µm for *Trichodesmium* and using spherical shape with diameter of 5 µm for *C. watsonii*, 1 µm for *Synechococcus*, 0.8 µm for picoeukaryotes, 0.5 µm for *Prochlorococcus*, and 0.3 µm for HB [58]. The C contents were estimated from the literature: 30 ± 28 ng C *Trichodesmium* filament^−1^ [76], 255 ± 43, 36 ± 6 and 2590 ± 730 fg C cell^−1^ for *Synechococcus*, *Prochlorococcus,* and pico-eucaryotes, respectively [77] and 12.4 ± 6.3 fg C cell^−1^ for HB (both HNA and LNA) [78], except for *C. watsonii* that was measured in this study.

*n/a* no data available.

^a^in mol C trichome^−1^ for *Trichodesmium*.

^b^in µm² filament^−1^.

Finally, by accounting for the abundance of each group in situ, we calculated the group-specific contribution to the bulk Fe uptake (Eq. S3a). For this, we also considered a group named ‘Others’, defined as the contribution of the remaining biological Fe uptake (Eq. S3b) that can be attributed to organisms also assimilating Fe, but that were not abundant enough to be sorted, such as diatoms, nanoeukaryotes and bacteria attached to particles. Note that these data are subject to high variability due to the error propagation of both SF- and GS-experiments resulting in high standard deviations. At S10-B, the major contributors to the bulk Fe uptake were *Trichodesmium* (51 ± 41%), followed by HB (19 ± 17%), while the contribution of *C. watsonii* was very low (<1%) (Fig. 3b). At S10-H, the contribution of *Trichodesmium* and *C. watsonii* together was the highest (17 ± 8% for *Trichodesmium* and 32 ± 17% for *C. watsonii*), followed by that of *Prochlorococcus* and that of HB (12.6 ± 8% and 13 ± 7%, respectively). At S10-A, the contribution of ‘Others’ dominated the Fe demand (49 ± 68%), followed by that of *Prochlorococcus* (34 ± 21%), *Trichodesmium* (8 ± 5%) and the contribution of both *C. watsonii* and HB was low (<5%) compared to that of the two other stations. Overall, at all stations, the contribution of *Synechococcus* and picoeukaryotes was low (<5%).

## Discussion

### Specific Fe uptake rates: focus on diazotrophs

In the literature, biological Fe uptake rates are usually reported for the total microbial community [57, 81–83], for size fractions [60, 84] (Table S4), but information on the group-specific Fe uptake remain scarce [35, 85] and often limited to picoplankton. Here, we combined ^55^Fe tracer methods with cell-sorting and give new insights into group-specific in situ Fe uptake rates. It allowed us to quantify for the first time in situ Fe uptake rates for *C. watsonii* and to compare them with those of *Trichodesmium* and the rest of the microbial community. The cell-specific Fe uptake rates of *C. watsonii* and *Trichodesmium* reported here were higher by factors of 3–16 and 42 to >400 than those of all other sorted (non-diazotrophic) organisms, respectively (Fig. 3a). This may be explained by their large cell size (~4–7 µm in diameter for *C. watsonii*, ~900 µm long, ~5 µm width for *Trichodesmium*) compared to that of pico-phytoplankton and bacteria (<2 µm), and by the unique Fe burden imposed by N_2_ fixation [13, 17, 24, 25, 86].

*Trichodesmium* cellular Fe uptake rates (Fig. 3b) were 11 to 70-fold higher than those of *C. watsonii* and when normalized to their surface area, rates were still higher than those of *C. watsonii*. This likely reflects the higher Fe requirements for *Trichodesmium*, independently of their larger size, as well as the low Fe requirements of *C. watsonii* as a consequence of the regulation of their metalloenzyme inventories during the day (Saito 2011). However, the surface area normalized rates of *C. watsonii* were in the same range or lower than these unicellular organisms (Table 2). This result may reflect that *C. watsonii* cells are larger than the picoplankton which results in lower surface to volume ratio and higher surface diffusion layer that likely unfavored *C. watsonii* to acquire Fe [30].

**Fig. 4:**
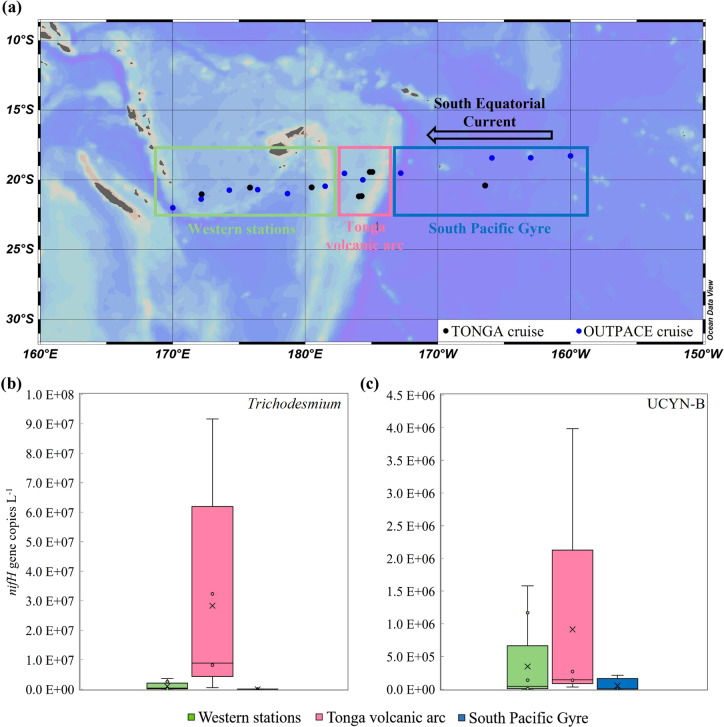
Patterns of *Trichodesmium* and *C. watsonii* distribution in the South Pacific. **a** Map of the WTSP showing the sampling stations from the OUTPACE cruise (2015, 10.17600/15000900, blue dots) and TONGA cruise (2019, 10.17600/18000884, black dots). The green frame delimits the Western stations, the pink frame delimits the Tonga volcanic arc stations and the blue frame delimits the South Pacific Gyre stations. Surface (5 m) abundances of **b** UCYN-B and **c** *Trichodesmium* at Western stations (*n* = 8 for *Trichodesmium*, *n* = 9 for UCYN-B), Tonga volcanic arc (*n* = 5 for *Trichodesmium* and UCYN-B), and South Pacific Gyre (*n* = 4 for *Trichodesmium* and UCYN-B) from the OUTPACE cruise [73] and from the TONGA cruise (Unpublished), presented as boxplots (center line = median, box limits = first and third quartiles, whiskers = data min and max).

To reflect the Fe demand of diazotrophs (i.e. the intracellular Fe content), Fe:C quotas (µmol Fe mol C^−1^) were calculated from Hudson et al. [51] (Eq 3) using our Fe uptake rates and growth rates from the literature (Table S5).The Fe:C quotas for *Trichodesmium* were 2 to 12 times higher than those of *C. watsonii*, and they were in the same range as previously reported [22, 28]. Although this difference is attenuated compared to the cellular Fe uptake rates, it confirms that *Trichodesmium* requires more Fe per unit C biomass than *C. watsonii*. This may reflect both the singular capacity of *C. watsonii* to intracellularly recycle Fe [24], thus requiring less ambient Fe but it may also reflect the remarkable capacity of *Trichodesmium* to take up and assimilate a wide range of Fe substrates from highly bioavailable inorganic Fe to strong binding ligands such as siderophores or particles [26, 27, 87, 88]. As a comparison, Fe:C quotas for *Synechococcus*, *Prochlorococcus* and HB were 2–52 times lower (on average over all stations) than those averaged for the two diazotrophs (Table S5). These calculated Fe:C quotas should be considered cautiously as we used estimated growth rates from the literature but overall, these results suggest that both studied diazotrophs have a high Fe demand compared to that of the non-diazotrophic plankton, and that *Trichodesmium* requires more Fe per unit of C than *C. watsonii*, which seems better adapted than *Trichodesmium* to thrive in Fe-limited environments [24]. This feature is clearly reflected by the biogeographical distribution of diazotrophs observed during this cruise, where *C. watsonii* was ~32-fold and *Trichodesmium* 5-fold more abundant (*p* < 0.05 non-parametric Mann–Whitney test) in the vicinity of the Tonga Arc where multiple hydrothermal sources fuel the water column with dFe [44, 47, 89] compared to stations located further west (Table S1). This led to N_2_ fixation rates significantly higher (by 3.3 times, *p* < 0.05 non-parametric Mann–Whitney test) in that area (53 nmol N L^−1^ d^−1^ on average over the Tonga arc stations) compared to those measured at western stations (16 nmol N L^−1^ d^−1^). We must remain cautious about these conclusions drawn on a 5-stations dataset, and we therefore combined diazotrophs abundance data (qPCR) from this cruise with those from a previous cruise (18 stations in total) that took place along a similar longitudinal transect [73] during the same season (Fig. 4a). This combined dataset confirms that *Trichodesmium* and *C. watsonii* are 3 to 30 times more abundant near the Tonga arc, where dFe concentrations average 1.6 nM over the photic layer, compared to downstream western stations (dFe concentrations 0.8 nM) (Fig. 4b, c) [44, 48]. In comparison, in the low dFe waters of the South Pacific Gyre (0.3 nM on average over the photic layer [44, 90]), *Trichodesmium* abundances are almost nil and *C. watsonii* abundances are even lower (by 5-fold) than at western stations (Fig. 4b, c). These findings are in accordance with the very high N_2_ fixation rates reported around the Tonga arc (~1000 µmol N m^−2^ d^−1^), the medium rates at western stations (~500 µmol N m^−2^ d^−1^) and the low rates in the South Pacific Gyre (~90 µmol N m^−2^ d^−1^) [43]. Combined together, these observations suggest that the Tonga volcanic arc is an intense N_2_ fixation area, likely due to high ambient dFe concentrations able to support the high Fe demand of diazotrophs, although Fe speciation could also play a significant role. The positive correlations between Fe uptake and N_2_ fixation rates found in this study, and between N_2_ fixation and dFe concentrations in the WTSP [43], support this hypothesis. Other environmental factors are suspected to shape the diazotroph distribution in the WTSP: when the high DIP (~100 nM), low NO_3_^−^ waters from the South Pacific Gyre are advected west of the Tonga trench by the South Equatorial Current in Fe-rich and warm (>25 °C) waters, all environmental conditions are fulfilled for diazotrophs to bloom extensively [43, 46].

Interestingly, bulk Fe uptake rates and dFe concentrations were higher (by 2.7- and 2.4-fold on average, respectively) in the Tonga arc stations (S10 stations) compared to those at western stations (S11 and S12). This suggest that the concentration of dFe, and potentially its speciation, likely influenced the different rates between the two regions but further physico-chemical measurements of Fe speciation are needed to determine how the nature of the complexing ligands influenced dFe bioavailability. DIP concentrations also differed between the two regions (below quantification limit in western stations, and low but still detectable, 0.06 µM in the Tonga arc stations), and may limit Fe uptake rates at western stations. However, the similar contribution (~30–38%) of P-normalized Fe uptake rates of each size-fraction over all stations suggest that the pico-, the nano- and the microplankton community responded homogeneously to different environmental conditions. In the quest to examining the main contributors to the Fe uptake in the WTSP, this result reinforces the need to measure Fe uptake at a group-specific level.

### The role of picoplankton on the microbial Fe demand

By accounting on average for 53% (or 32% when normalized to biomass) of the bulk Fe uptake rates over all stations (Table 1), picoplankton (0.2–2 µm) was, with diazotrophs, a major contributor to total Fe uptake. Within the picoplankton, *Prochlorococcus* and HB generally contributed predominantly to the total Fe uptake (24–63% for *Prochlorococcus*, 9–31% for HB). Their fast growth rates (~1.9 d^−1^ for HB [91], and ~0.4 d^−1^ for *Prochlorococcus* [92]) compared to that of *C. watsonii* (mostly retained on the 2–10 µm fraction, growth rate ~0.05 d^−1^) together with their high abundance and unique capacity to take up strongly complexed Fe from siderophores [93**–**95], might explain why these two populations contribute substantially to bulk Fe uptake, despite having low cell-specific uptake rates.

Within the HB consortium, high (HNA) and low (LNA) nucleic acid content bacteria are distinguished [96]. HNA are thought to be larger, more active and have higher growth rates than LNA [96**–**98], which may explain the higher cellular Fe uptake rates measured for HNA than for LNA (Fig. 3a, Table 2). At S10-A, LNA were more abundant than HNA and the contribution of HB to total Fe uptake was the lowest among all stations, highlighting the potentially important role of HNA in Fe uptake within HB.

Finally, the picoeukaryote cellular Fe uptake rates were higher than those of the non-diazotrophic cyanobacteria, but their contribution to the bulk uptake was overall very low (0.5–1.5%), likely due to their low abundances (~360 cells mL^−1^). When considering the S.A. specific rates, they also acquired Fe at faster rates than all other non-diazotrophic unicellular cyanobacteria (Table 2), suggesting that in situ Fe substrates were more rapidly assimilated by picoeukaryotes compared to cyanobacteria. Fe uptake kinetics of inorganic Fe are similar for cyanobacteria and eukaryotes of the same size, while eukaryotes generally acquire Fe-bound complexes more efficiently than cyanobacteria [31, 99]. Hence, Fe speciation data for this region are needed to further understand the success of picoeukaryotes in acquiring Fe.

### Evaluation of dFe bioavailability in WTSP waters

Recent studies have used the apparent Fe uptake rate constant *k*_in-app_ to compare the ability of different plankton species to take up Fe from highly diverse Fe complexes [34, 59]. They suggest that a dFe bioavailability proxy can be assessed by the uptake rate constant normalized to cell surface area (*k*_in-app_/S.A.) for Fe-limited plankton species in the natural environment.

**Fig. 5:**
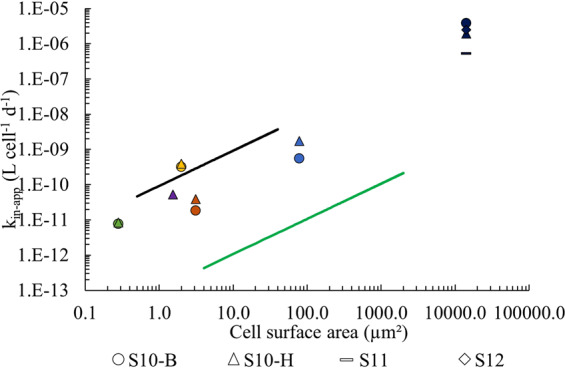
Calculated apparent Fe uptake rate constant k_in-app_ (Eq. S4) at S10-B, S10-A and S10-H for *Trichodesmium* (dark blue marks), *C. watsonii* (blue marks), *Synechococcus* (orange marks), *Prochlorococcus* (purple marks), picoeukaryotes (yellow marks) and HB (green marks) as a function of their cellular surface area (S.A.) in a log-log plot. dFe bioavailability envelope from [34] is boundaried by the unchelated inorganic iron Fe’ (black line) and the strong complex FeDFB (green line) Each station is labeled by a unique symbol.

The apparent Fe uptake rate constant *k*_in-app_ (Eq. 3) was calculated for *Trichodesmium, C. watsonii*, *Synechococcus*, *Prochlorococcus*, picoeukaryotes and HB against their respective estimated S.A (Fig. 5; Table 2) and we added the ‘dFe bioavailability envelope’ proposed by Lis et al. [34] for Fe-limited eukaryotic phytoplankton. This envelope is delimited by the highly bioavailable inorganic Fe as its upper boundary and by the very low bioavailable Fe complexed to a siderophore (DFB) at its lower boundary. Our data are the first to report *k*_in-app_ for cyanobacteria and HB in their natural habitat. Most of our values fall within the ‘dFe bioavailability envelope’, suggesting that this envelope can be utilized not only for eukaryotes but also for cyanobacteria and HB. Furthermore, the *k*_in-app_ shows a rough proportionality to the S.A. of the cyanobacteria and HB. This facilitates normalization of the *k*_in-app_ by S.A and allows for an assessment of the dFe bioavailability proxy *k*_in-app_/S.A in this area [59]. On average between all organisms, *k*_in-app_/S.A at S10-B and S10-H were 9.4 ± 12 × 10^−11^ and 7.1 ± 7.4 × 10^−11^ L µm^−2^ d^−1^, respectively. The high variability we obtained may result from the wide size range of the sorted organisms (from 0.5 µm² cell^−1^ for HB to >14 × 10^3^ µm² filament^−1^ for *Trichodesmium*). On average, *k*_in-app_/S.A at S10 (8.2 × 10−11 L µm^−2^ d^−1^) is 34-fold lower than for inorganic Fe (2.4 × 10^−9^ L µm^−2^ d^−1^) and 29-fold higher than for FeDFB (2.4 × 10^−12^ L µm^−2^ d^−1^) [34]. This suggests that the dFe bioavailability of this hydrothermally-impacted seawater was higher than model Fe-siderophore complexes and approached the highly available inorganic Fe. While equilibration of inorganic ^55^Fe with in situ ligands likely occurred during the experiment, our *k*_in-app_ could have been impacted by the organisms acquiring inorganic Fe upon its addition. It is also possible that the photodegradation of the ligands during daylight released inorganic Fe that influenced our rates [59, 100], yet our observations are consistent with the dFe bioavailability investigated for Fe-limited eukaryotes [61]. Finally, the proportionality between *k*_in-app_ and S.A. of cyanobacteria and HB reinforces the hypothesis that Fe-limited microorganisms can be used to probe the bioavailability of dFe [59].

## Conclusions

In this study, we provide a group-specific view of Fe uptake by the microbial community in the WTSP. We show that ~33% of the in situ Fe uptake is by the diazotrophs *C. watsonii* and *Trichodesmium*, despite being numerically less abundant in surface waters compared to the rest of the microbial community. Yet, the picoplankton, particularly HB and *Prochlorococcus*, is the major contributor to biological uptake of the dFe pool. Our study also reveals that poor dissolved inorganic P and Fe-rich waters impacted by hydrothermal fluids seem to favor diazotrophs, which have a high Fe demand as suggested by their high Fe:C ratios, compared to those of the non-diazotrophic plankton. This study therefore demonstrates that Fe sources other than Fe-rich mineral dust [101] (namely shallow hydrothermal sources) influence the biogeographical distribution of diazotrophs in the ocean, which may also be useful for modelers. Finally, by introducing this group-specific Fe uptake approach for in situ populations, our study opens a variety of possibilities for research in environmental microbiology. With the ability to differentiate and separate individual functional groups within microbial communities, techniques as we applied with Fe may be used for other biogeochemically-relevant trace metals to examine the ecophysiological roles of functional groups in their natural habitat.

## Supplementary information

Supplementary Materials
