# Supplementary material for: Assessing the contribution of diazotrophs to microbial Fe uptake using a group specific approach in the Western Tropical South Pacific Ocean
Source: ISME Commun. 2022 Apr 27;2:41. doi: 10.1038/s43705-022-00122-7 (PMC9723570; doi:10.1038/s43705-022-00122-7)
Supplement: Supplementary file 1 — Supplementary Materials [file 43705_2022_122_MOESM1_ESM.docx]

**Supplementary Materials**

**Contents**

**Trace metal clean techniques** (page 2)

**Figure S1** (page 3)

**Figure S2** (page 4)

**Blank calculations** **and Fe uptake rates equations for SF- and GS-experiments, Figure S3** (page 5)

**Table S1** (page 7)

**Table S2** (page 8)

**Table S3** (page 9)

**Table S4** (page 10)

**Table S5** (page 11)

**References** (page 12)

**Trace metal clean techniques**

Incubation PC bottles were soaked for 1 week in alkaline detergent Decon 90 and rinsed five times with demineralized water followed by ultrapure MilliQ water rinse (3 times) into a trace metal clean laboratory (clean room, ISO5). They were then filled with 10% analytical grade HCl for one week followed by 5 rinses with Ultrapure MilliQ water and filled with 100 mL of 10% Suprapur (Merck) HCl for several weeks until use. Prior to sample collection for incubation experiments, the bottles were vigorously rinsed 3 times with surface seawater from the TMR.


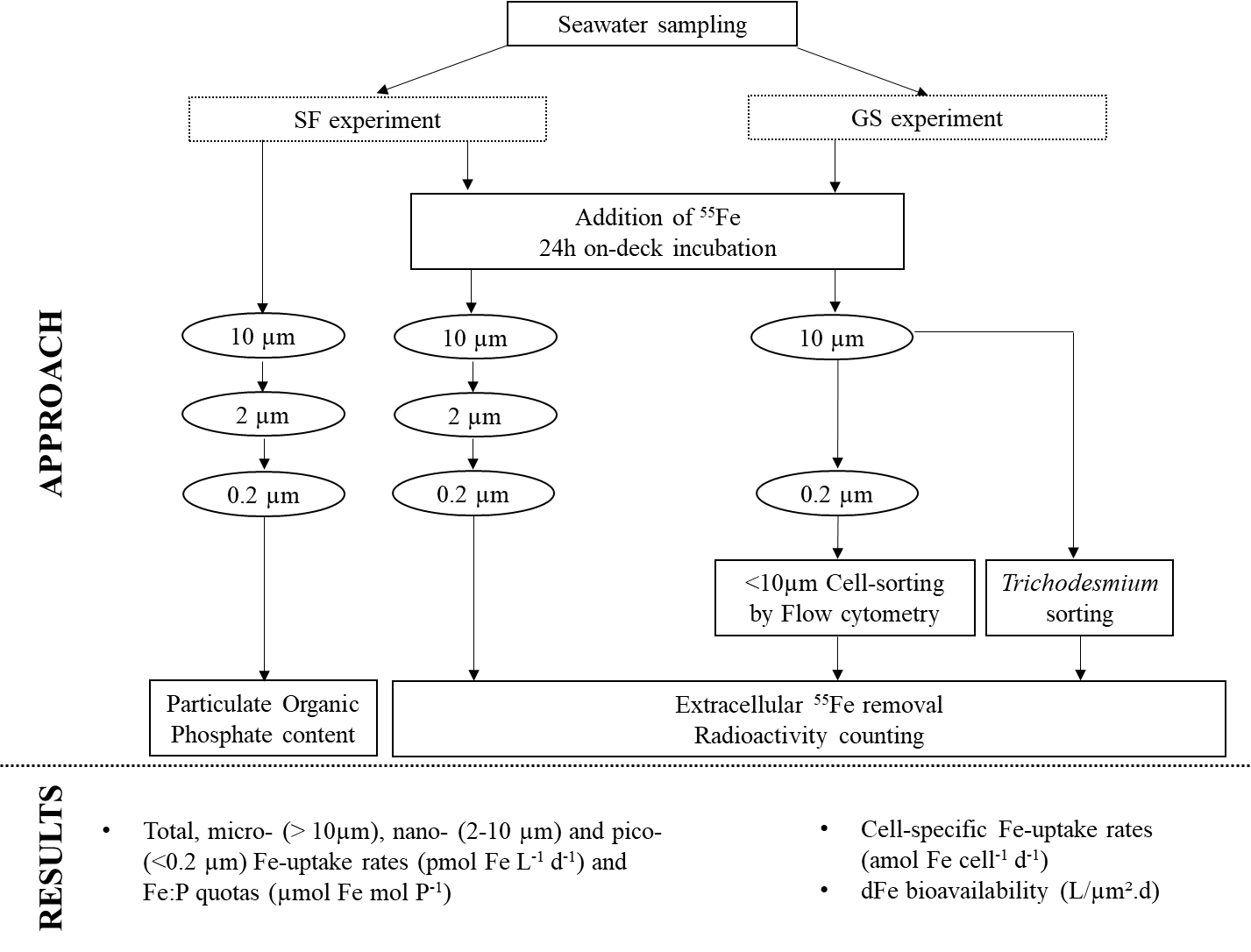


**Figure S1.** Schematic representation of size-fraction (SF) and group-specific (GS) experiments to determine Fe uptake rates by different members of the microbial community during the TONGA cruise. During the GS experiments, specific diazotrophic and non-diazotrophic groups of the microbial community were sorted.

| **a) 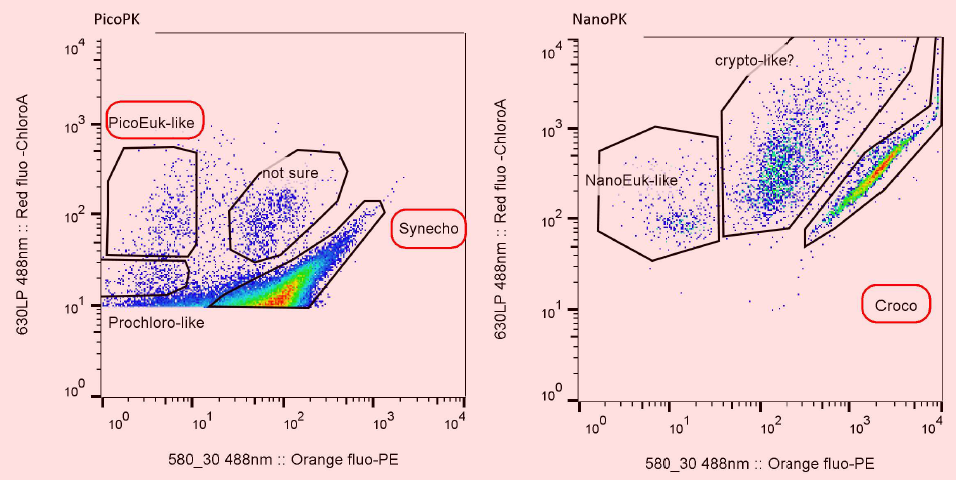** | 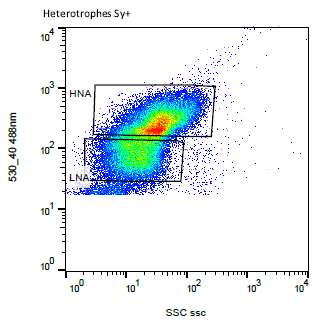 |
| --- | --- |
| **b)**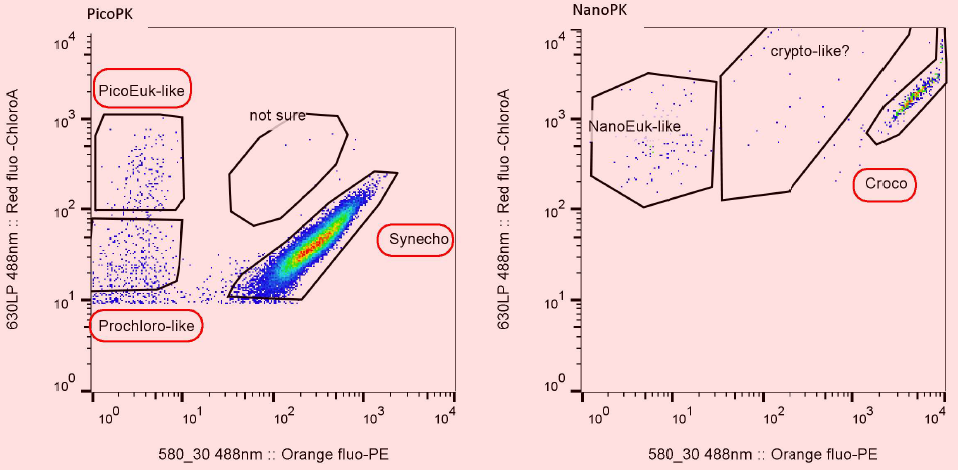 | 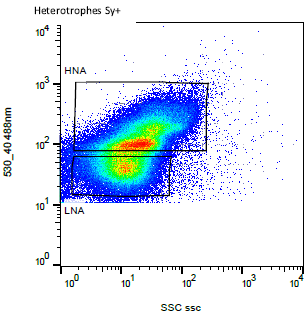 |
| **c)**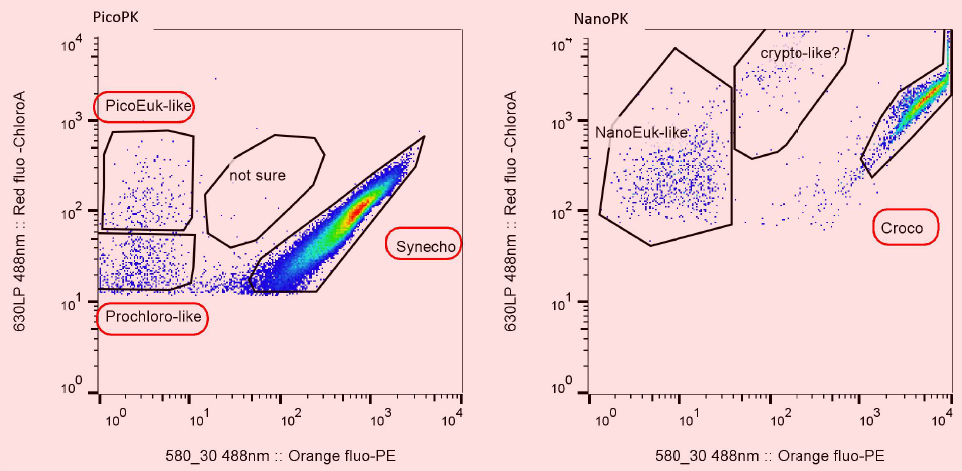 | 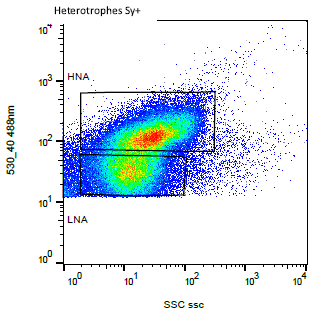 |
| **Figure S2.** Example of cytograms obtained during cell sorting by flow cytometry, showing clusters of *C. watsonii*, *Synechococcus*, *Prochlorococcus*, pico-eukaryotes and heterotrophic bacteria (HB) with high (HNA) and low (LNA) nucleic acid content at **a)** S10-B, **b)** S10-A and **c)** S10-H. | |

**Blank calculations** **and Fe uptake rates equations for SF- and GS-experiments** Size-

Size-Fractionation (SF) Experiment:

At each station, a control bottle was amended with formaldehyde (0.5% final concentration) to stop all biological activity and spiked 15 min later with the same amount of ^55^Fe and treated the same way as experimental bottles. The radioactivity for each control filter (A_SF Blank_) corresponds to the non-desorbing adsorption of ^55^Fe that is considered as background ^55^Fe uptake. The activity of the blank is subtracted from the activity on the SF-filter (A_SF filter_) to calculate intracellular ^55^Fe on the filter (in mol, Eq S1.a), which is injected in Eq S1.b to give the Fe uptake rates for each size fraction (ρ_pico_, ρ_nano_ and ρ_micro_ in mol Fe L^-1^ d^-1^):

$55Fe on filter=\frac{A_{Ffilter}-A_{Blank}}{Specific Activity of 55Fe source}$ **(Eq S1.a)**

$SF-Fe uptake rate : \rho_{SF}=\frac{55Fe on filter}{{t_{inc}\times V}_{inc}}\times\frac{\left[ dFe \right]seawater+[55Fe] added}{[55Fe] added}$ **(Eq S1.b)**

$Bulk Fe uptake rate: \rho_{Bulk}= \rho_{pico}+ \rho_{nano}+ \rho_{micro}$ **(Eq S1.c)**

Group-Specific (GS) Experiment:

For each sorted organism, we have plotted the number of disintegrations per minute (dpm) against the number of sorted cells. The y-intercept from the linear curve is considered as the abiotic blank (A_GS Blank_) (Table in Figure S3). The activity of the blank is subtracted from the activity of the filter containing the cells (A_cells_) to calculate the intracellular ^55^Fe uptake (in mol, Eq S2.a) which is injected in Eq S2.b to give group-specific Fe uptake rates (ρ_Tricho_, ρ*_C.Watsonii_*_,_ ρ_Syn_, ρ_Proch_, ρ_picoeuk_, ρ_HNA_ and ρ_LNA_, in mol Fe cell^-1^ d^-1^):

$55Fe on sorted organism=\frac{A_{Cell}-A_{GS Blank}}{Specific Activity of 55Fe source}$ **(Eq S2.a)**

$GS-cellular Fe uptake rates:\rho=\frac{55Fe on sorted organism}{{t_{inc}\times number of cells}}\times\frac{\left[ dFe \right]seawater+[55Fe] added}{[55Fe] added}$ **(Eq S2.b)**

Fe uptake rates of HB (ρ_HB_, in mol Fe cell^-1^ d^-1^) were calculated by using HNA and LNA specific uptake rates (ρ_HNA_ and ρ_LNA_) and their abundances (see Table S1), as described in Eq 2.c:

$HB cell specific Fe uptake rate:\rho_{HB}= \frac{\rho_{GS-HNA}\times abundance HNA+ \rho_{GS-LNA}\times abundance LNA}{abundance HNA+ abundance LNA}$ **(Eq S2.c)**

The contribution of each sorted organisms to the bulk Fe uptake is calculated as described in Eq S3.a (e.g. for *Trichodesmium*):

$GS-Contribution of Trichodesmium (\%)=100\times\frac{\rho_{Tricho}\times abundance of Trichodesmium}{\rho_{Bulk}}$ **(Eq S3.a)**

The contribution of ‘others’ is calculated as described in Eq S3.b:

$Others contribution \left( \% \right)=1-\sum Contribution of sorted organisms$ **(Eq S3.b)**

The apparent Fe uptake rate constant k_in-app_ (in L cell^-1^ d^-1^) was calculated using the equation from (1) (e.g. for *Trichodesmium*): $k_{in-app}=\frac{\rho_{Tricho}}{{[dFe]}_{seawater}}$ **(Eq S4)**

|  | \|  \| Blank  (dpm) \| S10-H  (dpm) \| \| --- \| --- \| --- \| \| *Trichodesmium* \| 1016 \| 3460 \| \| *Crocosphaera Watsonii* \| 489 \| 3491 \| \| *Synechococcus* \| 333 \| 952 \| \| *Prochlorococcus* \| 64 \| 144 \| \| Pico eukaryotes \| 50 \| 108 \| \| HB \| 99 \| 298 \| |
| --- | --- | --- | --- | --- | --- | --- | --- | --- | --- | --- | --- | --- | --- | --- | --- | --- | --- | --- | --- | --- | --- | --- |

**Figure S3.** Example of blank calculation for pico-eukaryotes: the y-intercept corresponds to the remaining ^55^Fe when no cells of pico eukaryotes uptake Fe. On the righten side, the blank for each sorted organism and the activity of the filter of the sorted organism at S10-H are reported.

**Table S1**. Summary of environmental conditions in the study area at 5m depth: N_2_ fixation and primary production rates, nutrients concentrations and *in situ* abundances and C biomass of the major planktonic groups sorted by flow cytometry in this study. LQ: quantification limit

|  | **S10 - B** | | | **S10 - A** | **S10 - H** | | | **S11** | | | **S12** | | |
| --- | --- | --- | --- | --- | --- | --- | --- | --- | --- | --- | --- | --- | --- |
| **N_2_ fixation rates** (nmol N L^-1^ d^-1^) | 64.8 | ± | 15.5 | - | 41.5 | ± | 9.8 | 17.4 | ± | 9.8 | 15.0 | ± | 9.4 |
| **Primary Production** (µmol C L^-1^ d^-1^) | 2.49 | ± | 0.73 | - | 0.55 | ± | 0.004 | 1.18 | ± | 0.43 | 0.68 | ± | 0.07 |
| **NO2** (µM) | < LQ=0.01 | | | < LQ=0.01 | < LQ=0.01 | | | < LQ=0.01 | | | < LQ=0.01 | | |
| **NO3** (µM) | < LQ=0.05 | | | < LQ=0.05 | < LQ=0.05 | | | < LQ=0.05 | | | < LQ=0.05 | | |
| **PO4** (µM) | 0.06 | | | 0.05 | 0.06 | | | < LQ=0.02 | | | < LQ=0.02 | | |
| **Si(OH)4** (µM) | 1.15 | | | 1.05 | - | | | - | | | 0.8 | | |
| **DOC** (µM) | 77 | | | 76 | 70 | | | 80 | | | 72 | | |
| ***Trichodesmium***  (filament L^-1^) | 2637 | | | 2687 | 1851 | | |  | - |  |  | - |  |
| ***Trichodesmium***  (*nifH* gene copies L^-1^)  (µmol C L^-1^) | 32 x 10^6^  6.6 | | | - | 8.8 x 10^6^  6.7 | | | 4.0 x 10^6^  4.6 | | | - | | |
| ***C. Watsonii***  (*nifH* gene copies L^-1^)  (µmol C L^-1^) | 2.7 x 10^5^  0.2 | | | 7.5 x 10^5^*  0.55 | 4.0 x 10^6^  2.9 | | | 6.6 x 10^4^  0.05 | | | 4.8 x 10^4^*  0.04 | | |
| ***Synechococcus***  (cell mL^-1^)  (µmol C L^-1^) | 9 946  0.21 | | | 4 417  0.09 | 8 181  0.17 | | | 3 165  0.07 | | | 2 502  0.05 | | |
| ***Prochlorococcus***  (cell mL^-1^)  (µmol C L^-1^) | 44 378  0.13 | | | 17 524  0.05 | 52 100  0.16 | | | 26 699  0.08 | | | 6 610  0.02 | | |
| **Pico eukaryotes**  (cell mL^-1^)  (µmol C L^-1^) | 346  0.08 | | | 498  0.11 | 249  0.05 | | | 282  0.06 | | | 422  0.09 | | |
| **HNA** (cell mL^-1^)  (µmol C L^-1^) | 558 821  0.58 | | | 375 773  0.53 | 332 306  0.34 | | | 417 954  0.43 | | | 522 056  0.54 | | |
| **LNA** (cell mL^-1^)  (µmol C L^-1^) | 375 642  0.39 | | | 513 081  0.92 | 281 560  0.31 | | | 290 091  0.30 | | | 466 071  0.48 | | |
| *Abundance measured by flow cytometry (in cell L^-1^) | | | | | | | | | | | | | |

**Table S2.** Detailed data of the SF-Experiment: DPM (in triplicates) measured for each size fraction and blanks, ^55^Fe added, *in situ* dFe concentrations and Fe uptake rates calculated using Equations S1.

| **Stations** | **Filter porosity**  *µm* | **DPM** | **specific activity** *dpm/mol* | **^55^Fe added** *nM* | **dFe in situ** *nM* | **Fe uptake rate** *nmol Fe/L.d* |
| --- | --- | --- | --- | --- | --- | --- |
| S10-B | 10 | 10506 | 1.38E+16 | 0.20 | 0.49 | 4.62E-03 |
| S10-B | 2 | 1997 | 1.38E+16 | 0.20 | 0.49 | 7.28E-04 |
| S10-B | 0.2 | 3410 | 1.38E+16 | 0.20 | 0.49 | 1.44E-03 |
| S10-B | 10 | 3022 | 1.38E+16 | 0.20 | 0.49 | 1.03E-03 |
| S10-B | 2 | 5278 | 1.38E+16 | 0.20 | 0.49 | 2.30E-03 |
| S10-B | 0.2 | 21547 | 1.38E+16 | 0.20 | 0.49 | 1.01E-02 |
| S10-B | 10 | 13163 | 1.38E+16 | 0.20 | 0.49 | 5.89E-03 |
| S10-B | 2 | 9509 | 1.38E+16 | 0.20 | 0.49 | 4.32E-03 |
| S10-B | 0.2 | 42735 | 1.38E+16 | 0.20 | 0.49 | 2.03E-02 |
| Blank S10-B | 10 | 859 |  |  |  |  |
| Blank S10-B | 2 | 476 |  |  |  |  |
| Blank S10-B | 0.2 | 398 |  |  |  |  |
| S10-A | 10 | 4421 | 1.38E+16 | 0.20 | 1.56 | 5.13E-03 |
| S10-A | 2 | 5511 | 1.38E+16 | 0.20 | 1.56 | 6.69E-03 |
| S10-A | 0.2 | 14804 | 1.38E+16 | 0.20 | 1.56 | 1.84E-02 |
| S10-A | 10 | 3759 | 1.38E+16 | 0.20 | 1.56 | 4.29E-03 |
| S10-A | 2 | 5355 | 1.38E+16 | 0.20 | 1.56 | 6.49E-03 |
| S10-A | 0.2 | 5682 | 1.38E+16 | 0.20 | 1.56 | 6.86E-03 |
| S10-A | 10 | 1551 | 1.38E+16 | 0.20 | 1.56 | 1.49E-03 |
| S10-A | 2 | 1887 | 1.38E+16 | 0.20 | 1.56 | 2.09E-03 |
| S10-A | 0.2 | 4897 | 1.38E+16 | 0.20 | 1.56 | 5.87E-03 |
| Blank S10-A | 10 | 377 |  |  |  |  |
| Blank S10-A | 2 | 236 |  |  |  |  |
| Blank S10-A | 0.2 | 271 |  |  |  |  |
| S10-H | 10 | 9825 | 1.38E+16 | 0.18 | 0.35 | 3.91E-03 |
| S10-H | 2 | 6121 | 1.38E+16 | 0.18 | 0.35 | 2.42E-03 |
| S10-H | 0.2 | 15504 | 1.38E+16 | 0.18 | 0.35 | 6.23E-03 |
| S10-H | 10 | - | 1.38E+16 | 0.18 | 0.35 | - |
| S10-H | 2 | - | 1.38E+16 | 0.18 | 0.35 | - |
| S10-H | 0.2 | - | 1.38E+16 | 0.18 | 0.35 | - |
| S10-H | 10 | 8768 | 1.38E+16 | 0.18 | 0.35 | 3.49E-03 |
| S10-H | 2 | 10668 | 1.38E+16 | 0.18 | 0.35 | 4.26E-03 |
| S10-H | 0.2 | 23751 | 1.38E+16 | 0.18 | 0.35 | 9.56E-03 |
| Blank S10-H | 10 | 147 |  |  |  |  |
| Blank S10-H | 2 | 129 |  |  |  |  |
| Blank S10-H | 0.2 | 109 |  |  |  |  |
| S11 | 10 | 5959 | 1.38E+16 | 0.16 | 0.41 | 2.25E-03 |
| S11 | 2 | 974 | 1.38E+16 | 0.16 | 0.41 | 1.82E-04 |
| S11 | 0.2 | 2533 | 1.38E+16 | 0.16 | 0.41 | 1.06E-03 |
| S11 | 10 | 4287 | 1.38E+16 | 0.16 | 0.41 | 1.39E-03 |
| S11 | 2 | 3017 | 1.38E+16 | 0.16 | 0.41 | 1.30E-03 |
| S11 | 0.2 | 11481 | 1.38E+16 | 0.16 | 0.41 | 5.64E-03 |
| S11 | 10 | 8084 | 1.38E+16 | 0.16 | 0.41 | 3.33E-03 |
| S11 | 2 | 2867 | 1.38E+16 | 0.16 | 0.41 | 1.23E-03 |
| S11 | 0.2 | 6337 | 1.38E+16 | 0.16 | 0.41 | 3.01E-03 |
| Blank S11 | 10 | 1576 |  |  |  |  |
| Blank S11 | 2 | 474 |  |  |  |  |
| Blank S11 | 0.2 | 470 |  |  |  |  |
| S12 | 10 | 10347 | 1.38E+16 | 0.16 | 0.24 | 3.39E-03 |
| S12 | 2 | 5799 | 1.38E+16 | 0.16 | 0.24 | 1.91E-03 |
| S12 | 0.2 | 10992 | 1.38E+16 | 0.16 | 0.24 | 3.75E-03 |
| S12 | 10 | 1907 | 1.38E+16 | 0.16 | 0.24 | 4.32E-04 |
| S12 | 2 | 2289 | 1.38E+16 | 0.16 | 0.24 | 6.83E-04 |
| S12 | 0.2 | 12152 | 1.38E+16 | 0.16 | 0.24 | 4.16E-03 |
| S12 | 10 | 11992 | 1.38E+16 | 0.16 | 0.24 | 3.96E-03 |
| S12 | 2 | 2157 | 1.38E+16 | 0.16 | 0.24 | 6.37E-04 |
| S12 | 0.2 | 1793 | 1.38E+16 | 0.16 | 0.24 | 5.33E-04 |
| Blank S12 | 10 | 672 |  |  |  |  |
| Blank S12 | 2 | 337 |  |  |  |  |
| Blank S12 | 0.2 | 271 |  |  |  |  |

**Table S3.** Detailed data of the GS-Experiment: DPM for each sorted organisms and blanks, ^55^Fe added, *in situ* dFe concentrations and cellulare Fe uptake rates calculated using Equations S2.

| **Stations** | **Sorted organisms** | **DPM** | **specific activity** *dpm/mol* | **^55^Fe added** *nM* | **dFe in situ** *nM* | **Fe uptake rate** *amol Fe/cell.d* |
| --- | --- | --- | --- | --- | --- | --- |
| S10-B | *Trichodesmium* | 7571 | 1.38E+16 | 0.20 | 0.49 | 32.510 |
|  | *C. Watsonii* | 1876 | 1.38E+16 | 0.20 | 0.49 | 0.464 |
|  | *Synechoccocus* | 488 | 1.38E+16 | 0.20 | 0.49 | 0.015 |
|  | *Prochloroccocus* | - | 1.38E+16 | 0.20 | 0.49 | - |
|  | Pico eukaryote | 334 | 1.38E+16 | 0.20 | 0.49 | 0.271 |
|  | HNA | 190 | 1.38E+16 | 0.20 | 0.49 | 0.005 |
|  | LNA | 184 | 1.38E+16 | 0.20 | 0.49 | 0.002 |
| S10-A | *Trichodesmium* | 1319 | 1.38E+16 | 0.20 | 1.56 | 5.974 |
|  | *C. Watsonii* | 715 | 1.38E+16 | 0.20 | 1.56 | 0.361 |
|  | *Synechoccocus* | 911 | 1.38E+16 | 0.20 | 1.56 | 0.005 |
|  | *Prochloroccocus* | 192 | 1.38E+16 | 0.20 | 1.56 | 0.376 |
|  | Pico eukaryote | 129 | 1.38E+16 | 0.20 | 1.56 | 0.557 |
|  | HNA | 133 | 1.38E+16 | 0.20 | 1.56 | 0.001 |
|  | LNA | 116 | 1.38E+16 | 0.20 | 1.56 | 0.001 |
| S10-H | *Trichodesmium* | 3460 | 1.38E+16 | 0.18 | 0.35 | 13.487 |
|  | *C. Watsonii* | 3491 | 1.38E+16 | 0.18 | 0.35 | 1.213 |
|  | *Synechoccocus* | 952 | 1.38E+16 | 0.18 | 0.35 | 0.027 |
|  | *Prochloroccocus* | 144 | 1.38E+16 | 0.18 | 0.35 | 0.036 |
|  | Pico eukaryote | 108 | 1.38E+16 | 0.18 | 0.35 | 0.271 |
|  | HNA | 467 | 1.38E+16 | 0.18 | 0.35 | 0.005 |
|  | LNA | 129 | 1.38E+16 | 0.18 | 0.35 | 0.001 |
| S11 | *Trichodesmium* | 4636 | 1.38E+16 | 0.16 | 0.41 | 4.020 |
| S12 | *Trichodesmium* | 24657 | 1.38E+16 | 0.16 | 0.24 | 15.025 |

**Table S4**. Bulk Fe uptake rates, POC-normalized Fe uptake rates and contribution of the picoplanktonic fraction (0.2–2 µm) to the bulk Fe uptake in this study compared to published studies of natural surface waters in other oceanic regions.

| **Reference** | **Oceanic region** | **dFe** | **Bulk Fe uptake** | | **POC-normalized Fe uptake** | **0.2 - 2 µm fraction** | |
| --- | --- | --- | --- | --- | --- | --- | --- |
|  |  | (nM) | (pmol Fe  L^-1^ d^-1^) | | (µmol Fe  mol C^-1^ d^-1^) | (pmol Fe  L^-1^ d^-1^) | (% of total) |
| **This study** | WTSP | 0.24-1.56 | 6.5–19 | | 1.2–3.5 | 2.8–10.6 | 43–63 |
| *Ellwood et al. 2020* (2) | SO (Subantarctic zone) | 0.02–0.06 | 11–27 | |  | 6.7–17.3 | 20–45 |
| *Fourquez et al. 2020* (3) | SO (Subantarctic zone) | 0.08 | 90–193 | | 48-102 | 20–46** | 60–73** |
| *Fourquez et al. 2015* (4) | SO (Kerguelen Plateau) | 0.06–0.38 | 19–40 | | 2.5–5.3 |  | 2 |
| *Strzepek et al. 2005* (5) | SO (SE of New Zealand) | 0.05 | 26–101 | |  |  | 1.4–4.3** |
| *Mioni et al. 2007* (6) | ESP | 0.08-0.3* | 10–567 | |  | 8–403* |  |
| *King et al. 2012* (7) | South West Pacific (NE of New Zealand) | 0.03-0.60 |  | |  | 5.1–14.2 | 17–60 |
| *rates for 0.2-1 µm fraction, **rates for 0.2-0.8 µm fraction | | | |  |  |  |  |

**Table S5.** Estimation of Fe:C quotas for diazotrophs (*Trichodesmium* and *C. Watsonii*), non-diazotrophic cyanobacteria (*Synechococcus* and *Prochlorococcus*) and HB, calculated from the equation (3) in (8). Calculations are based on the cellular Fe uptake rates measured in this study and growth rates (µ in d^-1^) from the literature. For diazotrophs, we estimated the growth rates based on cellular N_2_ fixation rates and cellular N content measured for *C. Watsonii* and *Trichodesmium* in the WTSP (9). With respect to the Fe-limitation conditions (when dFe <0.6 nM, phytoplankton is considered Fe-limited) suggested by (10), we considered stations S10-H, S10-B, S11 and S12 for the calculations of Fe:C quotas but we excluded S10-A (dFe concentration >0.6 nM)

|  |  | **µ** | **Quotas Fe:C** (µmol Fe mol C^-1^) | | | |
| --- | --- | --- | --- | --- | --- | --- |
|  | **References** | (d^-1^) | S10-B | S10-H | S11 | S12 |
| ***Trichodesmium*** | *Bonnet et al., 2018* (9) | 0.01 | 159 | 66 | 20 | 73 |
| ***C. Watsonii*** | *Bonnet et al., 2018*(9) | 0.05 | 13 | 34 |  |  |
| ***Synechococcus*** | *Worden and Binder* (11) | 0.37 | 1.9 | 3.5 |  |  |
| ***Prochlorococcus*** | *Worden and Binder* (11) | 0.36 | n/a | 37.8 |  |  |
| **HB** | *Van Wambeke et al., 2018* (12) | 1.92 | 1.8 | 3.0 |  |  |

**References**

1. Lis H, Shaked Y, Kranzler C, Keren N, Morel FMM. Iron bioavailability to phytoplankton: an empirical approach. ISME J. 2015 Apr 28;9(4):1003–13.

2. Ellwood MJ, Strzepek RF, Strutton PG, Trull TW, Fourquez M, Boyd PW. Distinct iron cycling in a Southern Ocean eddy. Nat Commun. 2020;11(1):1–8.

3. Fourquez M, Bressac M, Deppeler SL, Ellwood M, Obernosterer I, Trull TW, et al. Microbial Competition in the Subpolar Southern Ocean: An Fe–C Co-limitation Experiment. Front Mar Sci. 2020;6(January).

4. Fourquez M, Obernosterer I, Davies DM, Trull TW, Blain S. Microbial iron uptake in the naturally fertilized waters in the vicinity of the Kerguelen Islands: Phytoplankton-bacteria interactions. Biogeosciences. 2015;12(6):1893–906.

5. Strzepek RF, Maldonado MT, Higgins JL, Hall J, Safi K, Wilhelm SW, et al. Spinning the “ferrous wheel”: The importance of the microbial community in an iron budget during the FeCycle experiment. Global Biogeochem Cycles. 2005;19(4).

6. Mioni C, Pakulski J, Poorvin L, Baldwin A, Twiss M, Jeffrey W, et al. Variability in the in situ bioavailability of Fe to bacterioplankton communities in the eastern subtropical Pacific Ocean. Aquat Microb Ecol. 2007 Mar 13;46:239–51.

7. King AL, Sañudo-Wilhelmy SA, Boyd PW, Twining BS, Wilhelm SW, Breene C, et al. A comparison of biogenic iron quotas during a diatom spring bloom using multiple approaches. Biogeosciences. 2012 Feb 3;9(2):667–87.

8. Hudson RJM, Morel FMM. Distinguishing between extra- and intracellular marine phytoplankton. 1989;34(6).

9. Bonnet S, Caffin M, Berthelot H, Grosso O, Benavides M, Helias-Nunige S, et al. In-depth characterization of diazotroph activity across the western tropical South Pacific hotspot of N2 fixation (OUTPACE cruise). Biogeosciences. 2018 Jul 12;15(13):4215–32.

10. Shaked Y, Twining BS, Tagliabue A, Maldonado MT. Probing the Bioavailability of Dissolved Iron to Marine Eukaryotic Phytoplankton Using In Situ Single Cell Iron Quotas. Global Biogeochem Cycles. 2021 Aug 25;35(8):1–19.

11. Worden A, Binder B. Application of dilution experiments for measuring growth and mortality rates among Prochlorococcus and Synechococcus populations in oligotrophic environments. Aquat Microb Ecol. 2003;30:159–74.

12. Van Wambeke F, Gimenez A, Duhamel S, Dupouy C, Lefevre D, Pujo-Pay M, et al. Dynamics and controls of heterotrophic prokaryotic production in the western tropical South Pacific Ocean: Links with diazotrophic and photosynthetic activity. Biogeosciences. 2018;15(9):2669–89.
